# Epstein-Barr Virus-Induced Epigenetic Pathogenesis of Viral-Associated Lymphoepithelioma-Like Carcinomas and Natural Killer/T-Cell Lymphomas

**DOI:** 10.3390/pathogens7030063

**Published:** 2018-07-18

**Authors:** Lili Li, Brigette B. Y. Ma, Anthony T. C. Chan, Francis K. L. Chan, Paul Murray, Qian Tao

**Affiliations:** 1 Cancer Epigenetics Laboratory, Department of Clinical Oncology, State Key Laboratory of Oncology in South China, Sir YK Pao Center for Cancer and Li Ka Shing Institute of Health Sciences, The Chinese University of Hong Kong, Hong Kong, China; lili_li@cuhk.edu.hk (L.L.); brigette@clo.cuhk.edu.hk (B.B.Y.M.); anthonytcchan@cuhk.edu.hk (A.T.C.C.); 2 Institute of Digestive Disease and State Key Laboratory of Digestive Diseases, Department of Medicine and Therapeutics, The Chinese University of Hong Kong, Hong Kong, China; fklchan@cuhk.edu.hk; 3 School of Cancer Sciences, University of Birmingham, Birmingham B15 2TT, UK; p.g.murray@bham.ac.uk

**Keywords:** Epstein-Barr virus, CpG methylation, epigenetics, nasopharyngeal, gastric cancer, lung cancer, natural killer (NK)/T-cell lymphoma, pathogenesis

## Abstract

Cancer genome studies of Epstein-Barr virus (EBV)-associated tumors, including lymphoepithelioma-like carcinomas (LELC) of nasopharyngeal (NPC), gastric (EBVaGC) and lung tissues, and natural killer (NK)/T-cell lymphoma (NKTCL), reveal a unique feature of genomic alterations with fewer gene mutations detected than other common cancers. It is known now that epigenetic alterations play a critical role in the pathogenesis of EBV-associated tumors. As an oncogenic virus, EBV establishes its latent and lytic infections in B-lymphoid and epithelial cells, utilizing hijacked cellular epigenetic machinery. EBV-encoded oncoproteins modulate cellular epigenetic machinery to reprogram viral and host epigenomes, especially in the early stage of infection, using host epigenetic regulators. The genome-wide epigenetic alterations further inactivate a series of tumor suppressor genes (TSG) and disrupt key cellular signaling pathways, contributing to EBV-associated cancer initiation and progression. Profiling of genome-wide CpG methylation changes (CpG methylome) have revealed a unique epigenotype of global high-grade methylation of TSGs in EBV-associated tumors. Here, we have summarized recent advances of epigenetic alterations in EBV-associated tumors (LELCs and NKTCL), highlighting the importance of epigenetic etiology in EBV-associated tumorigenesis. Epigenetic study of these EBV-associated tumors will discover valuable biomarkers for their early detection and prognosis prediction, and also develop effective epigenetic therapeutics for these cancers.

## 1. Epigenetics in Cancer Pathogenesis—Beyond Genomics

Cancer development is a multiple-step process with multiple cancer gene abnormalities, which drives tumorigenesis through the disruption of key cellular signaling pathways [1,2,3]. Sequencing of various cancer genomes has revealed the importance of driver gene mutations in cancer pathogenesis, although involving only far fewer than 1% of all human genes [4,5]. It is remarkable that most driver mutations are from epigenetic regulatory genes, which are epigenetic modifiers acting as writers, readers, or erasers to regulate epigenetic signaling, gene transcription, and DNA repair and replication. For an example, *ARID1A*, as a subunit of the SWI/SNF (BRG1-associated factors) chromatin remodeling complex, has the highest mutation rate in human cancers [6,7], which plays a critical role in gene regulation and multiple tumorigenesis.

Meanwhile, epigenetic alterations, including promoter CpG methylation, histone modifications, chromatin remodeling, non-coding RNAs, and newly discovered RNA modifications, are reversible regulatory mechanisms, which enable cells to react and adapt quickly to external environmental stimuli, such as carcinogens, smoking, and oncogenic virus infection. Promoter CpG methylation and histone modifications can regulate critical cancer genes, including silencing tumor suppressor genes (TSG) and activating oncogenes. Abnormal promoter CpG methylation and histone codes occur frequently at the early stage of tumorigenesis, and thus could be valuable biomarkers for cancer diagnosis. The carcinogenesis model of chronic smoking exposure has demonstrated that smoking alone could induce somatic cells to a stem cell-like status, tightly controlled by epigenetic changes, facilitating further genetic changes to drive cells towards tumorigenesis [8]. It has been shown that engineered *p16^Ink4a^* promoter methylation is enough to cause early abnormal cell proliferation and tumor onset [9]. Thus, epigenetic alterations play a causal role in tumor initiation and progression, even prior to genetic mutations.

## 2. Unique Epigenetic Deregulation Induced by EBV during Tumorigenesis

EBV is a human herpesvirus with latent infection in >90% of the world population. EBV is strongly associated with several epithelial and lymphoid malignancies, including lymphoepithelioma-like carcinomas (LELC) of nasopharyngeal (NPC), gastric (EBVaGC), and lung tissues, as well as nasal natural killer (NK)/T-cell lymphoma (NKTCL), some Burkitt lymphomas, and Hodgkin disease [10,11,12]. EBV latent infection in tumor cells is associated with limited expression of viral proteins and RNAs, including latent membrane protein 1 (LMP1) and 2 (LMP2A), EBV-associated nuclear antigens (EBNAs), *Bam*HI-A rightward open reading frame 1 (BARF1), EBV-encoded small RNA (EBER), and *Bam*HI A RNA transcripts (BARTs), as viral latency I or II [13,14,15,16]. After infecting a single cell, EBV causes clonal proliferation of infected cells and leads to the development of pre-malignant lesion, playing a critical role in promoting the pathogenesis of EBV-associated tumors.

EBV-associated tumors are uncontrolled cell proliferative disorders derived from accumulated epigenetic and genetic abnormalities [10,17]. Although genetic susceptibility/alterations are crucial for the pathogenesis of EBV-associated tumors including NPC, EBV-induced epigenetic alterations in tumor cells are of equal, if not more, importance during EBV-associated tumorigenesis. Genome-wide CpG methylation (methylome) and transcriptome studies demonstrate a unique high-grade CpG methylation epigenotype of EBV-associated LELC, indicating a key role of EBV as an epigenetic driver in carcinoma pathogenesis, through establishing a distinct pathogenic program [18,19]. This high-grade methylation level is attributed to the modulation of host cell epigenetic machinery by EBV-encoded oncoproteins or RNAs, through epigenetic modifiers such as DNA methyltransferases (DNMTs), histone methyltransferases (HMTs), polycomb group (PcG) proteins and histone deacetylases (HDACs). For examples, upregulation of DNMTs (DNMT1, -3A, -3B) by LMP1 and LMP2A [20,21], upregulation of PcG protein Bmi-1 by LMP1 [22], as well as direct interaction of HDACs and polycomb repressive complex 2 (PRC2) with EBNA3 [23,24], have all been reported. Thus, EBV infection is able to modulate both DNA methylation-mediated transcriptional repression and heterochromatin formation through histone modifications during EBV-associated tumorigenesis.

## 3. Epigenetic Disruption/Activation of Cellular Genes Induced by EBV

Epigenetic alterations disrupt, (or activate), cancer genes including TSGs, (or oncogenes), involved in early cancer initiation and progression, through the regulation of cell transformation and malignant outgrowth [2,7,25]. Abnormal epigenetic modifications including mainly CpG methylation and histone modifications, which could be valuable biomarkers for the diagnosis and therapeutics of EBV-associated tumors.

### 3.1. CpG Methylation

CpG methylation is a well-studied epigenetic alteration associated with cancers. DNA methylation is reversible through active or passive processes. DNMTs, as master regulators of DNA methylation, are required for the maintenance of DNA methylation (5mC) and establishment of a new methylation pattern. Recent findings showed that 5-hydroxymethylcytosine (5hmC) is the sixth DNA base in mammalian genomic DNA [26]. Ten-eleven translocation (TET) family enzymes and isocitrate dehydrogenases (IDHs) mediate the demethylation conversion of 5mC to 5hmC.

Using genome-wide techniques, epigenomes (CpG methylomes) of EBV-associated tumors have been established, with the discovery of novel and known methylated genes involved in EBV-associated tumorigenesis. NPC methylomes have been established using methylated DNA immunoprecipitation coupled with microarrays (MeDIP-chip) [27], HumanMethylation450 (analyzing 485,000 CpG sites per genome) BeadChip [28,29], and MethylCap-sequencing [30]. EBVaGC methylomes have been profiled using Infinium HumanMethylation27 (analyzing 27,000 CpG sites per genome) and HumanMethylation450 BeadChips [31,32,33,34]. NK-cell lymphoma methylomes have been characterized using methyl-sensitive cut counting (MSCC) and reduced representation bisulfite sequencing (RRBS) platforms [35]. Epigenomes and transcriptomes of EBV-associated tumors display distinct biological patterns compared to their EBV-negative counterparts, with higher frequencies of gene methylation and >50% of gene expression and methylation affected by EBV infection [36,37,38].

A list of known and novel cancer genes inactivated by CpG methylation, involved in various cell signaling pathways, have been identified in EBV-associated tumorigenesis. For examples, promoter methylation silencing of *RASAL1* [39], *RASSF1A* [40,41], *DLC1* [42], and *DOK1* [43] in Ras and Rho GTPase signaling; methylation silencing of *PCDH10* [44,45], *PCDH17* [46], *SFRP1*, and *SFRP5* [27], *WNT5A* [47], *CHD11*, *DACT1* [27], and *ROR2* [47] in Wnt/β-Catenin signaling and epithelial-mesenchymal transition (EMT) regulation; methylation inactivation of *DLEC1* [48,49] and *PTPRK* [50] in STAT3 signaling; *UCHL1* [51] and *MGMT* [52] methylation linked to p53 and DNA repair signaling; *ZNF382* [53,54], *ZNF545* [55], *TET1* [56], and *PRDM5* methylation involved in chromatin and nuclear signaling; *p16* [57] methylation in cell-cycle regulation; as well as *ADAMTS18*, *CADM1* [58,59], and *DAPK1* [60] methylation related to cell apoptosis regulation. Specifically, *p16* silencing by epigenetic modulation occurs widely in the early stage of EBV-associated tumors, to overcome senescence for further oncogenic transformation and malignant proliferation. EBV infection precedes *E-cadherin* methylation, which was found in carcinoma tissues but not in dysplastic tissues [61], supporting the view that early epigenetic alterations induced by EBV are involved in EBV-associated pathogenesis. Therefore, more investigations should be performed to identify methylated novel cancer genes in EBV-associated tumorigenesis, verify their expression and methylation in tumor samples, and to assess their relationship to clinical features, as well as their potential as biomarkers.

Promoter CpG methylation of cancer genes are ubiquitously present in all human cancers but less in precancerous lesions, thus makes them as ideal biomarkers for cancer prognosis and prevention. Compared with other molecular markers such as mRNA and proteins, CpG methylation has many advantages in diagnosis application, including being stable, easily amplifiable and detectable, highly frequent, and non-invasive (directly from body fluids). Moreover, it occurs at the early stage of tumorigenesis. In EBV-associated tumors, some methylation markers and signatures have been identified, such as methylation of *PCDH10* [44,45], *TET1* [56], *WIF1* [62], and *DLEC1* [48,49] as early markers; *ZNF382* [53,54] methylation as a metastasis marker; *p16* [63,64], *RASSF1A* [40] and *WNT5A* [65] methylation as EBV-positive infection markers; *PTPRK* [50] methylation as a prognosis marker for NKTCL. Further investigations are thus needed for the discovery of more epigenetic biomarkers, especially at the early stage of EBV-associated malignancies.

### 3.2. Histone Modifications

Histones modification, as one of the epigenetic features, is involved in the regulation of chromatin structure and gene transcription. Its deregulation leads to cellular transformation and cancer progression [66]. Histone modifications include acetylation (-ac), methylation (-me), phosphorylation, ubiquitination, and sumoylation. Histone modifications regulate the accessibility of DNMTs, PcG complex proteins, and transcription factors, also as a link between DNA methylation and promoter activity. For example, histone H3 trimethylation of lysine 9 (H3K9me3) and histone H3 lysine 27 trimethylation (H3K27me3) are normally correlated with transcriptional repression, while H3K27ac and H3K4me3 are linked with active promoters.

Histone modifications regulate both EBV viral gene and host cell gene expression, to finely modulate EBV infection and EBV-induced tumorigenesis [67,68]. Histone deacetylation is correlated with the transcriptional repression of LMP1, BZLF1, and EBNA3C, as well as EBNA2 silencing, to regulate EBV latency [69]. LMP1 drives the expression of host cancer-promoting genes through activating poly(ADP-ribose) polymerase (PARP) and decreasing repressive H3K27me3 modification [70]. Histone modification is thus critically involved in EBV-mediated epigenetic reprogramming, which could be a therapeutic target for EBV-associated tumors.

## 4. EBV-Encoded Viral microRNAs and EBV-Regulated Host-Cell microRNAs

MicroRNAs (miRNAs), as another epigenetic regulatory mechanism, are also critically implicated in the development of EBV-associated neoplasms. EBV-encoded miRNAs [71,72,73] regulate host cell biology and microenvironment, contributing to cell proliferation, migration, and even the immune evasion of EBV [74,75,76]. EBV-encoded miRNAs are mainly composed of two groups: the BHRF1 miRNA cluster (miR-BHRF1-3) and BART miRNA cluster (miR-BART1-22), with simultaneous expression and multiple biological functions [73,77]. EBV-encoded miRNAs are transcribed and processed using the host miRNA apparatus. These miRNAs interfere with multiple cell signaling pathways and host immune surveillance, through targeting both viral and cellular mRNAs. For examples, four miR-BARTs directly target LMP1 [78], miR-BART22 targets LMP2A [79], and miR-BART20-5p targets BZLF1 and BRLF1 [80]. Multiple immune molecules are also directly regulated by miR-BHRF1s and miR-BARTs, such as IFN-γ, IRF3/IRF7, and IL-6/IL-10/IL-12B, etc. [75].

On the other hand, a series of host cell miRNAs have been shown to be regulated by EBV proteins [81], such as upregulation of miR-146a, miR-10b, miR-21, miR-29b, miR-34a, and miR-155 by LMP1 and LMP2A [82,83,84]; upregulation of miR-146a by BARF1 [85]; upregulation of let-7a and miR-127 by EBNA1; upregulation of miR-21 by EBNA2; downregulation of the miR-183-96-182 cluster, miR-15a, miR-1, miR-203, and miR-204 by LMP1; and downregulation of miR-34a and miR-146a by EBNA2 [86,87]. These cellular miRNAs further act as tumor suppressors or oncogenes.

Both viral and host-cell miRNA types are coordinately regulated to help EBV to escape host immune surveillance and contribute to EBV-associated tumorigenesis. In-depth functional and mechanistic investigations of these miRNAs during EBV pathogenesis would be a promising future direction for EBV research, and may also provide valuable biomarkers for EBV-associated tumors.

## 5. Epigenetic Implication to the Therapeutics of EBV-Associated Tumors

The unique feature of epigenetic therapy is the reversibility of epigenetic gene alterations, unlike the fixed hopeless oncogenic gene mutations. Mechanistically, epigenetic agents (such as Dacogen: 5-aza-deoxycytidine; Vidaza: 5-azacytidine) systematically affect cell regulatory programming, especially the abnormal program of stem cell-like (stemness) and drug-resistant properties of tumor cells, through correcting multiple cell signaling pathways including immune response signaling [88,89]. Cytotoxicity and off-target effect of epigenetic agents have been significantly diminished if used at low dosage [90,91,92], permitting widespread use of these agents for patients with hematological malignancies, as well as EBV-associated solid tumors. These agents have been tested in clinical trials for solid tumors [92], either alone or in combination with other drugs, with promising outcomes.

EBV-associated tumors are a good biological model system for testing demethylation drugs in vivo, as these tumors usually have fewer mutations but much more gene methylation. The first clinical trial using DNA methyltransferase inhibitor 5-azacytidine (Aza) in patients with EBV-associated tumors (NPC, Hodgkin and AIDS-related lymphomas) was conducted as early as 2000 [93]. After therapy, dramatic demethylation of EBV promoters (Wp, LMP1p, Zp, and Rp) was detected in these cancer patients. Immunohistochemistry further detected lytic Zta protein re-expression in NPC patients. Although no obvious clinical response was observed, this pilot trial provides experimental evidence for the feasibility of using demethylation drug for the treatment of EBV-associated tumors in vivo, especially when it is observed that NPC patients respond only modestly to immune therapy with PD-L1 antibodies [94].

In addition to viral proteins, detection of TSG reactivation in patient specimens treated with demethylation agents could provide a global view of the drug effect and guide future epigenetic therapy of EBV-associated tumors, including optimal drug dosage, and combinations with other therapeutic strategies.

## 6. Conclusions

**Figure 1:**
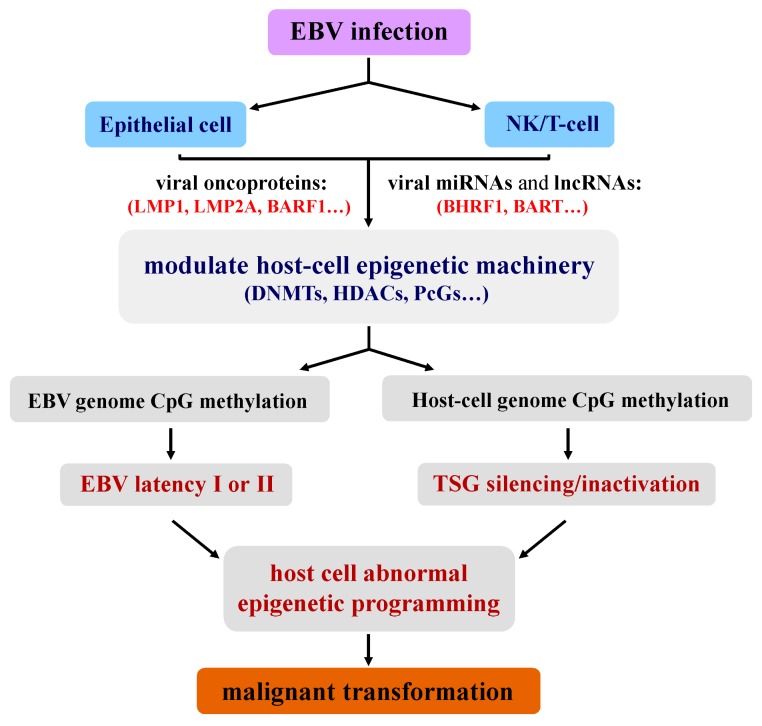
Model of EBV-induced epigenetic pathogenesis of viral-associated lymphoepithelioma-like carcinomas (nasopharyngeal carcinoma (NPC), EBV-associated gastric cancer (EBVaGC) and lung cancer) and natural killer/T-cell lymphoma (NKTCL). EBV-encoded oncoproteins, microRNAs (miRNAs) and long non-coding RNAs (lncRNAs) hijack cellular epigenetic machinery to reprogram viral and host-cell epigenomes, to establish an immune-evasive viral latency and oncogenic epigenetic program.

In the past 15 years, impressive advances have been made in elucidating the genomic and epigenomic changes of EBV-associated cancers, highlighting the central role of epigenetic mechanisms in the pathogenesis of EBV-associated tumors (Figure 1). Epigenetic alterations occur at the early cancer stage and throughout the whole disease stage, and thus are valuable biomarkers for early diagnosis and risk assessment. Furthermore, epigenetic therapeutics targeting epigenomic alterations have shown promising results in EBV-associated cancer management. Further profiling of the unique epigenetic signature of EBV-associated tumors and identification of epigenetic drivers related to EBV infection would provide greater molecular understanding of the pathogenesis of EBV-associated cancers, and would help to develop better biomarkers and effective therapeutic strategies.
